# Keeping Wavering Bonds: Deactivation-Induced Signaling by Reactive Electrophiles

**DOI:** 10.1021/acs.biochem.6c00173

**Published:** 2026-05-12

**Authors:** Marcus J. C. Long, Yaren Karakoç, Yimon Aye

**Affiliations:** † Department of Chemistry, 6396 University of Oxford, Oxford OX1 3TA, U.K.; ‡ Department of Pharmacology, University of Oxford, Oxford OX1 3QT, U.K.; § British Heart Foundation (BHF) Oxford Centre of Research Excellence (CRE), Oxford OX3 9DS, U.K.

## Abstract

Discovered ∼60years ago, the lipid metabolite 4-hydroxynonenal (HNE) is linked to a plethora of macromolecular targets and biological functions. For a molecule that weighs 156 Da and possesses a single H-bond donor, this is quite a feat. Despite its chemical simplicity, HNE contains an α,β-unsaturated aldehyde system, endowing it with the capability to react covalently with numerous biological functional groups and bestowing on it pleiotropic properties. Regardless of the specific entity engaging with HNE, it is covalent bond formation that has dominated thought on HNE behavior. Indeed, cells possess a flurry of detoxifying enzymes that convert HNE to less reactive chemicals lacking the α,β-unsaturated aldehyde. For instance, the cell can either reduce or oxidize the aldehyde within HNE, deactivating HNE’s chemical reactivity. Here, we discuss one of our recent papers that discovered that HNE can modify the detoxification enzyme, Cyp-33e1, in *Caenorhabditis elegans*, using a customized tissue-specific screen for HNE-sensor proteins. Consistent with the concepts of active site partitioning, HNE also emerged as a substrate of Cyp-33e1. We next discovered that HNE changed lipid storage in worms in a Cyp-33e1-dependent manner. We proposed that the product of Cyp-33e1 detoxifying HNE was responsible for this change in lipid storage and were able to show that 4-hydroxynonenoic acid (HNA), the product of Cyp-33e1 oxidation of HNE, causes this phenotype. We have dubbed this new signaling mode, “deactivation signaling”. It sets an important precedent for how the bioactivity of HNE is considered, and we discuss the ramifications of this result in this perspective.

## Graphical abstract

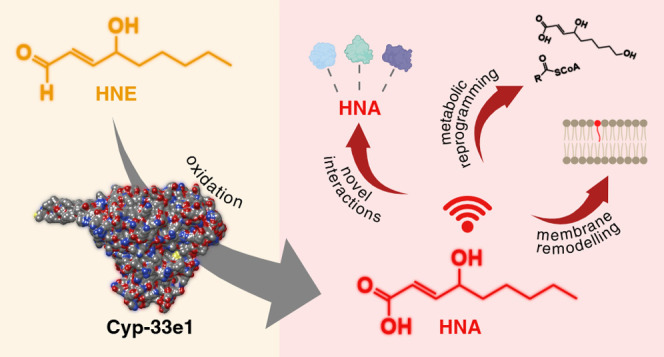

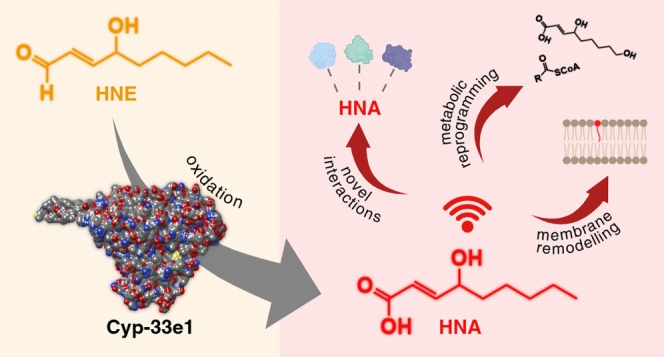

Since the work of Herman Esterbauer^1^ and later Earl Stadtman,^2,3^ 4-hydroxynonenal (HNE) has been considered to be a reactive signaling molecule. This simple, natural lipid-derived electrophile has been linked to a wealth of biological activities, from the beneficial to the deleterious.^4,5^ The basic concept that was proposed for the mode of action of this inherently reactive molecule was conjugate addition to nucleophilic species. Consistent with the aging field’s strong emphasis on DNA damage in the 1970s and 1980s,^6^ early work considered both DNA^7^ and proteins^8^ as important HNE targets (Figure 1). However, nowadays, in part because of the work by Stadtman,^9^ the focus is much more on specific protein targets.^1^

**Figure 1:**
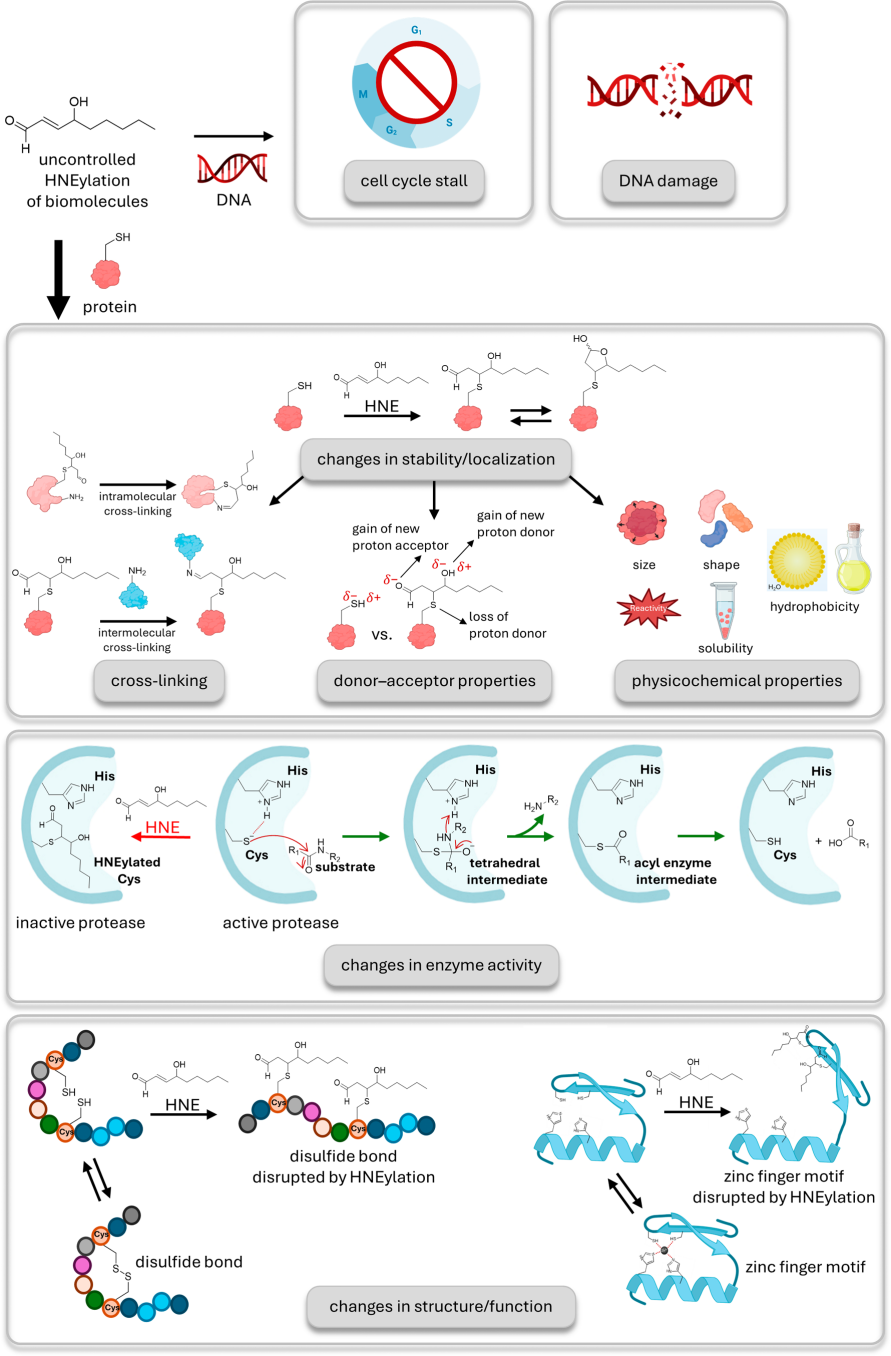
HNE can be conjugated to DNA and proteins with diverse outcomes. The first described conjugation event for HNE was with DNA through nucleophilic addition, leading to cell cycle arrest and DNA damage. In contrast, conjugation to proteins can have regulatory, favorable, as well as harmful effects. This adduction mainly takes place on cysteines through the sulfhydryl moiety. Here, the thiol group acts as a “soft” nucleophile. After adduction, the thiol of the cysteine becomes “lipidated”, manifesting effects on protein’s stability and localization, enzyme activity, or protein structure. The cysteine-HNE adduct can be in tautomeric equilibrium between the ring-opened and the ring-closed (hemiacetal) forms. Hereafter, only the ring-opened linear form is shown for simplicity. Changes in stability and localization can be directly attributed to the addition of the HNE molecule to the structure. First, the novel aldehyde group enables cross-linking to other proteins through amine, creating an imine bond, which is susceptible to further cross-linking. Another consequence of the adduction is the loss of proton donor thiol and gain of new proton acceptor aldehyde and donor hydroxyl groups, with ramifications for noncovalent interactions of the protein. Lipidation is expected to play a role in physical and chemical properties such as size, shape, availability for reactions, and solubility. Displayed on the middle panel, changes in enzyme activity can be induced by the loss of the thiolate, as in His–Cys proteases. The HNE modification takes place in the enzyme’s active site, rendering the enzyme unable to attack the protein substrate. Lastly, HNE adduction can result in structural changes. Here, two examples are given. Disulfide bonds are essential for secondary structure formation, and disruption of them due to cysteine-HNE adduction causes denaturation of the polypeptide chain. A similar effect is also seen on Zinc finger motifs. The structural integrity of the motif comes from the coordination of the Zn^2+^ ion (shown as a black sphere) with two histidine and two cysteine residues. Cysteine-HNE adduction leads to the destruction of this network.

HNE adduction to proteins occurs almost exclusively through reaction with protein cysteines; labeling likely occurs via the cysteine thiolate that exists in equilibrium with the protonated analog. However, as acid/base equilibrium establishes much faster than the labeling reaction proceeds—a situation referred to as Curtin-Hammett control^10^—and because as thiol p*K*_a_ decreases,^5^ thiolate reactivity decreases,^5^ relationships between thiolate in solution and reactivity are complex. Nonetheless, cysteine reacts preferentially because it is a “soft” nucleophile primed to interact with matched, “soft” electrophiles, like electron-deficient olefins.^11^ This process occurs several-fold faster than the equivalent process with amine-based nucleophiles,^5^ that are considered “hard” or more dominated by charge–charge interactions. A large body of evidence testifies to the fact that cysteine is a strategically important amino acid in protein structure. HNE-adduction induces a change from the free thiol (or potentially thiolate) form of cysteine to a lipidated version. The latter is larger, significantly more amphiphilic, and contains a carbonyl group that can undergo further modification, including cross-linking, significantly alters donor/acceptor, and physicochemical properties of the protein (Figure 1). For instance, the aldehyde of adducted HNE can form Schiff bases with lysine side chains. These links can be intramolecular, potentially distorting the preferred conformation. Otherwise, they can be intermolecular, which, by analogy to other known dimerization events, can cause gain-of-function signaling^12^ or sequestration of signaling proteins.^13^ As yet, to our knowledge, there have been relatively few studies on cross-linking-induced signaling at close to endogenous HNE concentrations. It can be speculated that intramolecular cross-linking could be linked to HNE’s proposed ability to destabilize proteins,^14^ for instance. Changes in association and hydrophobicity can also affect protein localization.^15^ In principle, HNE adduction at cysteine will not affect the protein isoelectric point significantly (as this alkylation event does not affect net charge). However, as interaction with lysine (or histidine) is possible, isoelectric point changes cannot be ruled out. HNE-adduction would necessarily affect enzyme activity should the labeled cysteine lie within an enzyme active site, as it can, for instance, in cysteine proteases^16^ and phosphatases^17^ (Figure 1). Moreover, there would be further important changes in structural integrity if the cysteine were part of a zinc finger or disulfide bond, among others (Figure 1).

Unsurprisingly, it was initially proposed that such modifications would negatively affect the stability of the labeled protein, leading to destabilization and degradation.^14,18^ Some evidence for this does exist,^19,20^ although the conditions used for these experiments were likely prone to identifying such behaviors due to the high concentration of electrophile used, as we discuss further below.

## It Is Difficult to Study “Natural” HNE Signaling

For many years, the study of HNE interacting with specific proteins was limited by our inability to recreate natural HNE signaling. Under conditions of nonpathological stress, HNE concentrations are likely strongly regulated and limited by detoxifying enzymes.^21^ When regulated increase in HNE production does occur, this likely happens in specific microdomains where its precursor lipids and oxidative stress coaccumulate.^22,23^ Unfortunately, experiments examining the direct effects of HNE were carried out either on purified proteins, which lack any of the detoxifying mechanisms of a cell or in cells or organisms bolus dosed (Figure 2A) with exogenous HNE.^5^ Neither really recapitulates what occurs under normal stress conditions. The augmentation of HNEylated proteins and other characteristic attributes of HNE upregulation have also been studied in diseased cells^24–26^ or during aging^27^ (Figure 2B). However, these experiments have difficulty in tracing cause and effect, and many of the detection methods are not necessarily precise, winnowing correlations between HNEylation and phenotypic manifestations, without even considering causation. Thus, how HNE modification of specific proteins fits into how the cell martials oxidative stress and aging and related phenotypic consequences was poorly defined for many years.^28^ Given HNE’s apparent proclivity to react with all thiols, many proposed that HNE generally wreaked havoc on the proteome, destabilizing proteins and/or inhibiting function overall, leading to cell death or genomic destabilization.^29^

**Figure 2:**
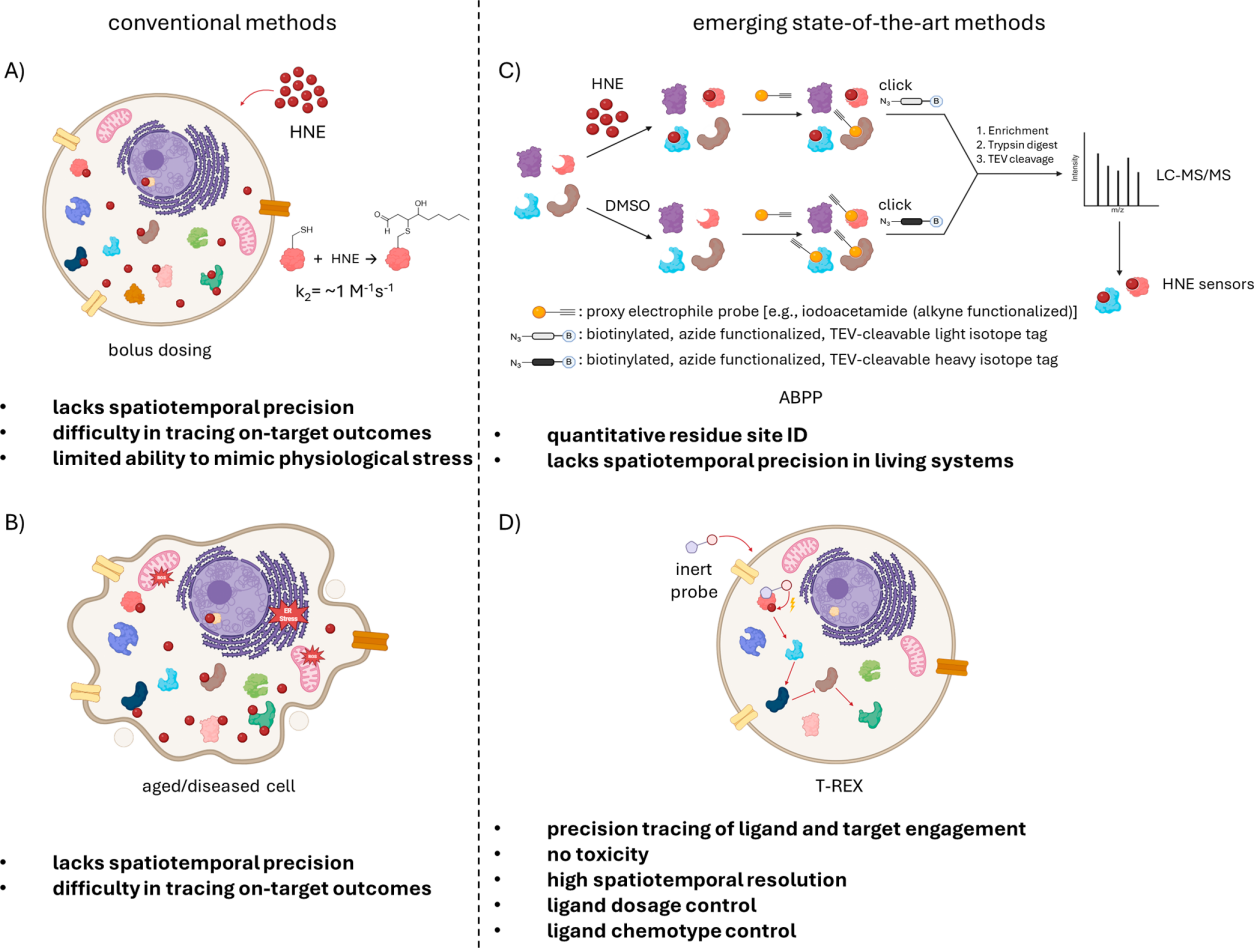
New chemical biology approaches enable the decoding of HNE signaling. (A) Established methods to study HNE adduction to protein cysteines were mainly based on treating the cells with bolus HNE. The second-order rate constant for the reaction of HNE with cysteine is 1.2 M^–1^ s^–1^. Excess treatment with HNE leads to promiscuous labeling of proteins and cannot precisely pin down privileged HNE sensors. It fails to recapitulate normal stress conditions where the amount and location of HNE signaling are controlled and cause toxicity. (B) HNE signaling has also been examined in aged or diseased cells where cellular stress is high, and HNE is upregulated. This environment is characterized by morphology changes, ER stress, and oxidative stress on mitochondria. (C) Shown here, competitive isoTOP-ABPP offers quantitative residue identification of HNE sensors. Two sets of lysates are treated with HNE or DMSO, followed by treatment with an alkyne-functionalized proxy electrophile probe, which binds to free cysteines in a nonspecific manner. Then, the HNE-treated group is labeled with the cleavable, biotin-bearing light isotope, whereas the DMSO-treated group is labeled with the heavy isotope via click reaction. Two samples are mixed, enriched using streptavidin resin, trypsin digested, and TEV cleaved. Tracking the absence of the probe, LC–MS/MS analysis captures HNElyated, reactive cysteines on HNE sensors. (D) T-REX (targetable reactive electrophiles and oxidants) allows detection of sentinel proteins taking part in HNE signaling without toxicity, with high spatiotemporal precision, and offers control of ligand dosage and type. It is an in vivo technique applicable to cell cultures, *C. elegans*, and zebra fish. Displayed here, cells are treated with the biorthogonal, photocaged HNE-containing probe. The probe covalently binds to the protein of interest. After treatment, HNE is uncaged via light in stoichiometric numbers and is able to result in a traceable signaling cascade.

Such a “blunderbuss” mechanism explains a large amount of the biology of HNE. However, it is noteworthy that the reaction of HNE with free cysteine or glutathione is not particularly fast (*k*_2_ = ∼1 M^–1^ s^–1^).^30,31^ And thus, HNE is certainly not as reactive as many of the indiscriminate electrophilic probes (e.g., iodoacetamide, quinone methides) widely used in indirect mapping of putative reactive cysteines and proximity profiling today that react with proteins at close to diffusion control (for instance, upper limit of *k*_2(carbene C–H insertion)_ = 10^7^ to 10^9^ M^–1^ s^–1^).^32,33^ This simple biophysical observation raises questions as to how indiscriminate HNE in cells actually is. Moreover, this model does not explain less intuitive results, such as hormesis effects; namely, that at low concentrations HNE can exert beneficial effects on cells and certain cellular processes.

## Chemical Biology Methods Increased Our Understanding of HNE Signaling

Some of the earliest works to experimentally address the indiscriminate reaction hypothesis were performed using activity-based protein profiling (Figure 2C), a method capable of assaying the reactivity of many different cysteines in a single experiment.^34^ To be scored as a hit in ABPP, HNE must achieve a high threshold occupancy on a specific protein target. By comparison of lysates treated with HNE versus a control sample, it was determined that relatively few proteins were, in fact, highly sensitive to HNE. Those proteins that were labeled efficiently by HNE actually differed from those that were labeled by another distantly related reactive carbon electrophile, 15-Deoxy-Δ12,14-prostaglandin J2, a cyclic enone. These data indicated another model, namely, that HNE reacts with specific sensor proteins whose labeling leads to downstream phenotypes observed under bolus dosing and, by extension, when HNE naturally builds up in cells. Seen through the eyes of this model, the large amount of proteins labeled under bolus HNE dosing may reflect an overwhelming of cellular defense mechanisms, consequential elevation of oxidative damage, or low percentage labeling of proteins that are not able to achieve a large occupancy and hence would not be scored by ABPP. However, whether such proteins could still serve as biologically relevant sensors remains an open question, as we will discuss later.

We also became interested in how the HNE functions. This was mainly borne out of the protein destabilization data,^19,20^ as it seemed to us to offer a chemical alternative to fluorescence-induced laser inactivation,^35,36^ which had once surfaced as a general means to destabilize targeted proteins. As shown in Figure 2D, we devised a method, dubbed T-REX,^37^ to label a specific protein with HNE, while not affecting cellular glutathione levels and not causing toxicity. This method deploys a Halo-fused protein of interest and a photocaged electrophile. Following light-driven uncaging, the liberated electrophile can react irreversibly and selectively with kinetically privileged endogenous protein cysteines within the vicinity of Halo. Using T-REX showed that labeling of select, such highly reactive sentinel proteins, including the known sentinel KEAP1,^37–42^ and other sentinel proteins we discovered, such as AKT3,^43,44^ was sufficient to cause biologically meaningful changes in signaling flux. It is important to appreciate that such changes were observed even though T-REX can only achieve substoichiometric labeling of the sentinel protein. We showed that this is in fact due to what appears to be a rather general property of privileged sensor proteins that they undergo dominant signaling mechanisms (e.g., dominant negative).^43–45^

We note that we have not observed targeted destabilization of HNEylated proteins in cells. Although this could be due to numerous factors, it seems likely to us that the destabilization of target proteins is likely linked to excessive HNEylation. In many cases, we do not believe that excessive HNEylation would be more likely to occur on the sentinel proteins that we discuss. Some of these proteins have numerous privileged HNEylation sites, although it is unclear how functionally coupled these are. Some of the models we have published would indicate that these are negatively coupled, meaning that sentinel proteins lose their heightened HNE sensitivity once labeled by HNE.

## Breaking the Bonds of General Models for HNE-Mediated Signaling

The above-mentioned models all contain one key and constant point: the reactivity of HNE, i.e., its ability to form a covalent bond with its target macromolecules, is the key to HNE’s signaling capabilities. The covalent bond, as we and others have discussed, confers extremely high effective “binding energy” between HNE and its targets while allowing the molecule to continue signaling until the labeled protein is degraded. We have postulated that proteins that react rapidly with HNE also contain an HNE-binding site, allowing a “liganded” binding interaction. Once HNE is appended to such target proteins, these low-affinity binding sites will be necessarily occupied due to proximity, maximizing the chances of signaling occurring. This mechanism potentially explains dominant signaling phenomena and why we have seen negative labeling cooperativity, where protein labeling by a specific reactive molecule does not reach a high percentage occupancy.

Given that covalent binding is so paradigmatic for HNE, we were quite surprised to find a new mechanistic paradigm for HNE signaling when we performed recent experiments in whole worms.^46^ Here we used a method, Localis-REX,^45–47^ that uses a photocaged, alkyne-tagged HNE, which can bind to the Halo-protein in conjunction with tissue-specific expression of the Halo protein. This setup is able to localize the buildup of alkyne-functionalized HNE in specific organs of the worm, allowing enrichment of HNE-sensor proteins in specific tissues. In this case, we investigated the pharynx, gut, or body wall muscles. In this way, using comparative proteomics, we identified specific proteins that are HNE-sensitive in specific tissues. Total proteome profiling experiments and comparison with single-cell RNA-seq data showed that these hits were not biased by the expression in specific tissues. One of the proteins we identified from this screen was a gut-specific HNE sensor, Cyp-33e1 (Figure 3A). We validated this protein and its human analogue, CYP2A6, as HNE sensors under various conditions. We also showed that CYP2A6, which emerged as the only one of the two proteins that could be purified successfully, was able to metabolize HNE to its acid form, HNA. HNEylation of CYP2A6 inhibited its enzymatic activity.

**Figure 3:**
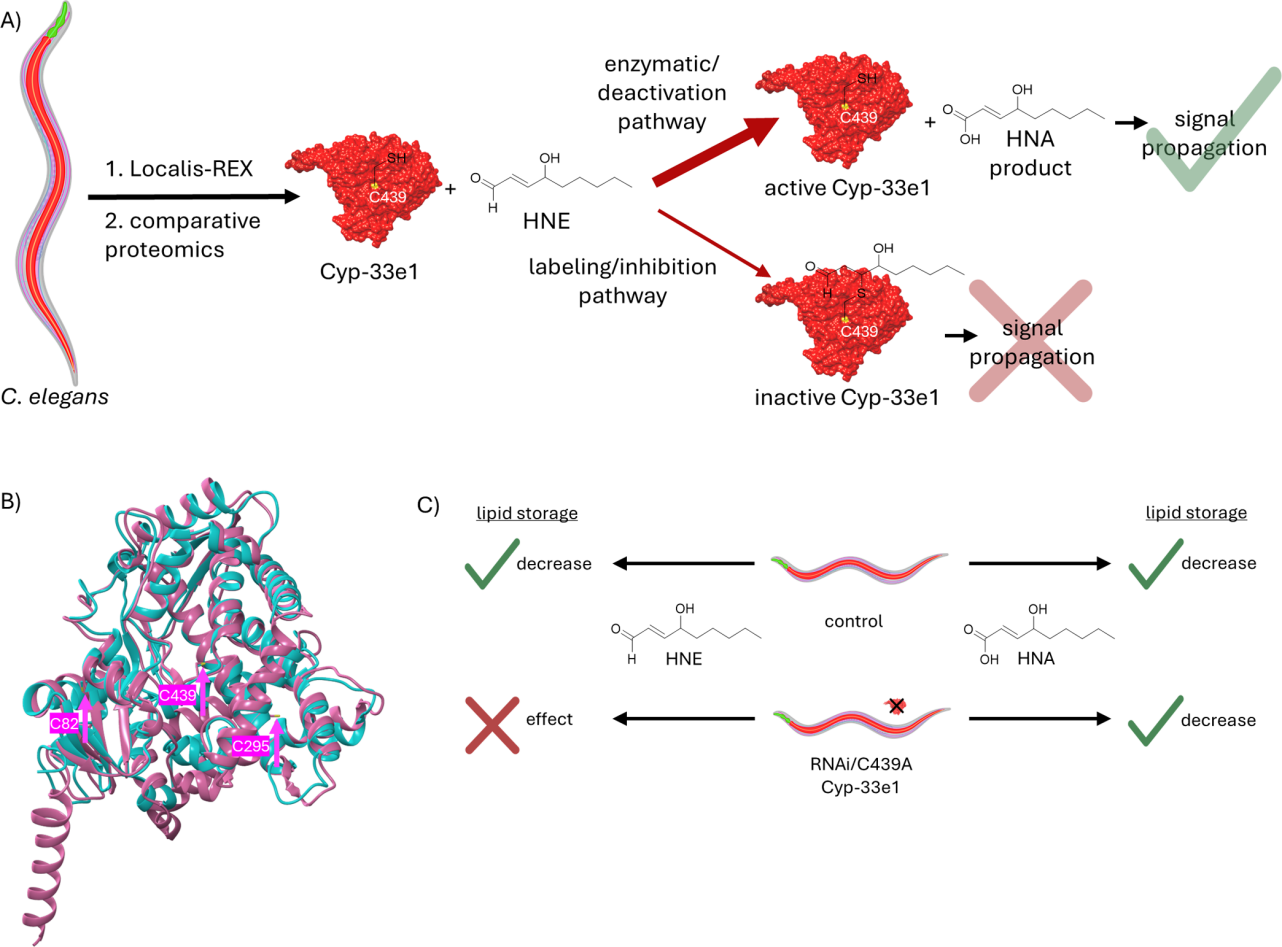
Gut-specific HNE sensor Cyp-33e1 is involved in deactivation signaling through HNA in *C. elegans*. (A) Proposed HNE signaling mechanism for Cyp-33e1 and its human ortholog CYP2A6. Different organs of *C. elegans* are sketched. Red: gut, green: pharynx, pink: body-wall muscle, red: gut. The nervous system and reproductive organs are shown transparent for simplicity. Localis-REX facilitates HNE release in specific locales, here in the gut, pharynx, and body wall muscle. Followed by comparative proteomics, it identified Cyp-33e1 as a gut-specific HNE sensor. Cyp-33e1 (shown here with a space-filling model from UCSF Chimera) and its human ortholog CYP2A6 are monooxygenases, metabolizing HNE to its carboxylic acid form, HNA. In the established mechanism, there are two signaling modes: enzymatic/deactivation and labeling/inhibition pathways. HNE acts as a substrate of the enzyme in the first pathway and a covalent inhibitor in the latter. Although a less reactive molecule compared to HNE (i.e., deactivated version of HNE), the main signaling is found to be through HNA. The inhibition of the enzyme is executed by HNElyation of C439 (highlighted as yellow), residing in the catalytic site. The labeling by HNE renders the enzyme inactive and halts the HNA signaling. (B) Overlay of Cyp-33e1 (AlphaFold AF-Q27482) and CYP2A6 (PDB 1Z10) models in ribbon presentation (pink: Cyp-33e1, cyan: CYP2A6). HNE sensing is accomplished by two cysteines in Cyp-33e1 (C439, C295) and CYP2A6 (C439, C82). C439 is conserved, taking part in enzyme activity, while C295 in worm and C82 in human take on additional HNE sensing roles. (C) HNE and HNA treatments reveal a novel HNA signaling mechanism. The first hypothesis is that HNE build-up in the gut will inhibit Cyp-33e1 and lead to a decrease in lipid storage. To test this, control and Cyp-33e1 silenced or catalytically inactive (C439A) worms were treated with HNE. The control group showed decreased lipid storage, while silencing Cyp-33e1 did not have an effect. This led to the conclusion that HNElyation of Cyp-33e1 is not the main signaling route in lipid storage deficiency. Accordingly, the second hypothesis is that the HNA, the metabolized form of HNE by Cyp-33e1, may be the main signaling molecule. Treatment of both control and Cyp-33e1-compromised worms showed a decrease in lipid storage, supporting the signaling role of HNA.

These basic data led us to propose that HNE buildup in the gut would inhibit Cyp-33e1 activity, potentially compromising HNE detoxification. Given HNE’s lipophilic nature and the fact that we had identified a sensing mode in the gut of the worm, we investigated how HNE treatment affected lipid storage. HNE treatment decreased lipid storage. We predicted that Cyp-33e1 silencing would exacerbate this phenotype. On the contrary, we found that Cyp-33e1-silenced worms were refractory to HNE-induced lipid storage deficiencies. Similar data were observed in KI strains for catalytically inactive Cyp-33e1. We thus came to the realization that although HNEylation of Cyp-33e1 may serve specific signaling roles, HNEylation of Cyp-33e1 was not involved in lipid storage defects incurred upon HNE treatment. We proposed, based on the available data, that it was in fact a product of Cyp-33e1 activity on HNE that caused the observed changes in lipid storage.

We indeed found that treating worms with HNA caused similar lipid storage deficiencies. HNA had these effects in control and Cyp-33e1-silenced worms (in contrast to HNE treatment; Figure 3C). We had thus established a new signaling paradigm, where deactivation of HNE through oxidation to the carboxylic acid leads to a new signaling molecule, HNA (Figure 3A). This mode of action is reminiscent of signaling carried out by several seemingly inert molecules, e.g., benzene, methanol, and many seemingly nonreactive drugs that nonetheless cause drug-induced liver injury (Figure 4). However, the principal difference is that in those cases metabolism by cytochrome enzymes causes the formation of a much more reactive product, e.g., benzeneoxide, formaldehyde, or acyl glucuronides. In the case of Cyp-33e1-orchestrated HNE signaling, the aldehyde group within HNE, which confers good acceptor properties to the conjugated olefin, is deactivated because the carboxylic acid unit bears a negative charge and hence is not as ready to sustain a nucleophilic attack. It should be noted that several studies have shown that the formation of HNA is more efficient than the formation of GSH-adducts in mammalian extracts.^48^

**Figure 4:**
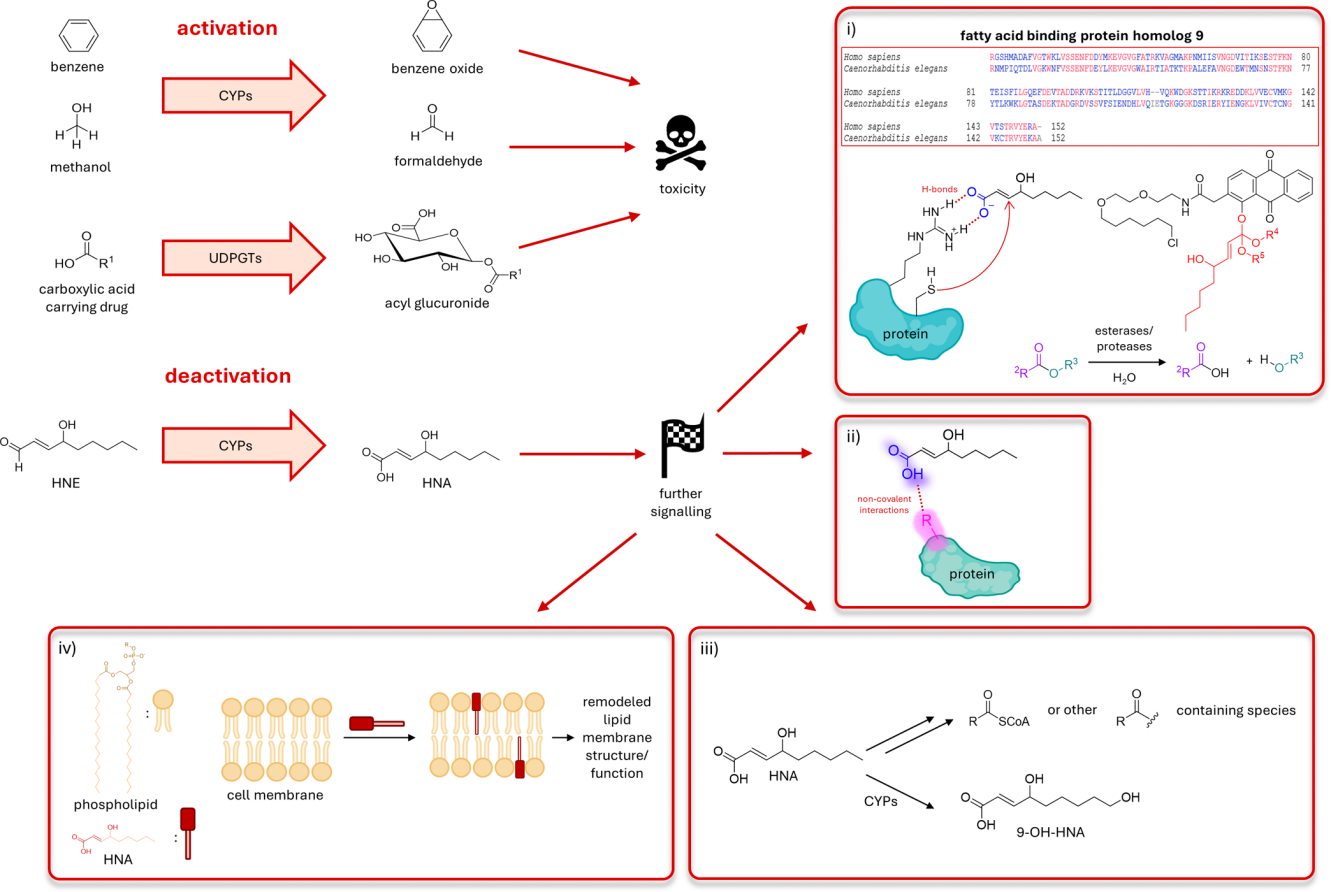
HNE’s deactivation to HNA can have various possible signaling outcomes. It is well established that inherently nontoxic molecules can be “activated” by certain enzymatic pathways to more reactive species. Examples of these are CYPs metabolizing benzene and methanol to benzene oxide and formaldehyde. Similarly, UDP-glucuronosyltransferases (UDPGT) in liver can turn carboxylic acid carrying drugs to acyl glucuronides, much more active molecules. Contrary to this, HNE’s “deactivation” to HNA by CYPs is a novel mechanism with further possible ramifications. Although less reactive, HNA can still react with proteins. One example of this would entail the protonated carboxylic acid residue docked onto a site in proximity to a reactive cysteine, facilitating the HNA’s addition to the cysteine’s thiol. Here (panel (i)), the protonated acid is shown H-bonding to an arginine residue. Above it, blast analysis shows that these cysteine and arginine residues are present in fatty acid binding protein homologue 9. In the comparison between the human and worm proteins, identical amino acids are colored red and nonidentical amino acids are colored blue. This signaling route can be studied using Localis-REX, which would require synthesis of a novel HNA photocaged probe. One way to do this is via an ester group. Since esters are susceptible to hydrolysis via esterases, it is challenging to produce a REX probe containing HNA. One promising way to achieve this is via an ortho-ester linkage. The photocaged probe with HNA bearing an ortho-ester is displayed in panel i. Red: HNA, black: targeting and photocaging units. Another way the cells can utilize HNA is through noncovalent interactions with proteins via the newly gained carboxylic acid group, which can allow stronger polarity compared to HNE’s aldehyde. Moreover, HNA can be metabolized to other signaling molecules such as acylCoA, other acyl containing groups, or 9-OH-HNA. Lastly, shown in panel iv, HNA can incorporate itself into the phospholipid bilayer of cellular membranes due to its polar head (dark red) and lipophilic tail (pink), similar to a phospholipid structure. Comparable to the effects of incorporation of proteins or small molecules such as cholesterol into the phospholipid membrane, HNA can have plenty of further implications for the cellular signaling modes.

## HNEylation Profiling Can Inform on Nonconjugative Signaling Modes

Before we progress to discussing the importance of deactivation-mediated signal transduction, it is worth discussing how we arrived at these conclusions. Cyp-33e1 was identified as an HNE sensor (i.e., a protein labeled by HNE) in a tissue-specific screen in live *C elegans*. HNE was identified as a substrate of the human analogue of Cyp-33e1, indicating that this protein can interact with HNE. Indeed, as we discussed above, proteins that are labeled by HNE effectively under low HNE concentrations are often those that have a putative HNE binding site. In vitro activity and inhibition studies using CYP2A6 further indicate that Cyp-33e1 undergoes partitioning between HNE oxidation and labeling. In this model, within the Cyp-33e1 active site, HNE undergoes two competing processes: first, enzymatic conversion to HNA and second, labeling of a reactive cysteine thiol in the active site [inhibitory (i.e., function/activity-coupled signaling)]. This sort of behavior is reminiscent of partitioning behaviors observed in the process of mechanism-based inhibition of several enzymes (including Cyps^49^), for instance, where partial turnover to product formation and enzyme inhibition occur.

We therefore think that HNE profiling methods, such as Localis-REX, are likely able to identify not only proteins that sense HNE through labeling but also those that are labeled by HNE through activity-coupled signaling modes. Activity-coupled signaling modes would likely only be able to achieve low HNE occupancy, as the prevailing action on HNE is metabolism/conjugation. This extends the remit of substoichiometric profiling methods and further gives another example of how substoichiometric labeling can be indicative of important biological functions. We note that such signaling modes are not detected by traditional profiling techniques. Activity-coupled signaling could be considered to be unique to Cyp-33e1. However, we note that there are at least 57 CYPs in humans,^50^ all of which may engage in analogous processes with reactive ligands. Moreover, many other proteins bind cofactors that could metabolize a bound HNE (or analog), including those involved in regulating sleep,^51^ immune and cell death signaling,^52^ and regulation of dNTP synthesis.^53^ Thus, there is already some evidence that activity-coupled signaling could be an overlooked mode of signaling.

## Biological Roles of Deactivation-Induced Signaling

At this point, we move toward more speculative aspects (Figure 4). It clearly remains to be established how the “deactivated” version of HNE, HNA, can elicit such an effect on lipid storage given that it is “inert”. We discuss several plausible possibilities and also how these could be experimentally addressed. We will focus the discussion on HNA and HNE, although we note that the discussion can likely extend to any reactive lipid and many other systems. Thus, these possibilities should serve as a springboard for following up on research data and discussion.

(i) What deactivation?

HNA is usually considered as a marker for metabolic detoxification of HNE.^54^ However, we note that HNA is not entirely unreactive, as it still contains an α,β-unsaturated carbonyl group, although this is highly deactivated. It is not inconceivable that some proteins could have the ability to react with HNA. This would require a protein that can protonate the carboxylic acid group and have a cysteine proximal to the olefin group when the carboxylic acid is docked to the proton donor. Indeed, the one known crystal structure of an HNE-bound protein complex depicts HNE adducted to the fatty acid acyl binding protein via a specific cysteine, C117.^55^ In this structure, the aldehyde (and likely the 4-OH group) is hydrogen bonded to a specific arginine, R126 (distances ∼4 Å). Blast analysis and structural modeling of specific worm isoforms indicate that both residues are present in one of the worm analogs, fatty acid binding protein homologue 9. Thus, this protein could afford a situation apposite for adduction to HNA, which could be more favorable than to HNE. Relatively little work has been done to understand how fatty acid-binding protein homologues function in *C elegans*, although one publication showed that homologue 5 regulates fat accumulation in worms.^56^ The data for other proteins was minimal.

Such a mode of action would be addressable by traditional proteomics techniques, including Localis-REX, although there is as yet not a validated means to photocage the carboxylic acid group applicable to REX technologies. This is in part because the acid is typically considered to be inert. One obvious method to photocage an acid would be to use an ester group, although these are prone to hydrolysis, and this arrangement would render the olefin electrophilic. Ortho-esters are the most likely candidates in this case.

On the other hand, as electrophile labeling of proteins is time-dependent, the half-life of each electrophile is relevant to their “covalent interactomes” and hence how they function biologically through covalent chemistry. GSH conjugation is well established to promote excretion from cells,^57^ making this metabolic process effectively end-point. However, the fate of HNA is much less well understood. Due to its lower inherent electrophilicity, HNA’s dwell time in cells is likely significantly longer than that of HNE and other reactive metabolites. This could allow HNA concentrations in cells to rise significantly, perhaps overcoming its low kinetic reactivity. Otherwise, the significantly increased half-life of HNA in cells would allow accumulation, possibly promoting its biophysical effects, independent of covalent labeling behaviors.

(ii) Less reactivity but new and strong noncovalent binding

Using an argument similar to that above, namely, that the donor and charge properties of HNA and HNE are different, we could also consider that HNA could interact noncovalently with a target protein more strongly than HNE (effectively leading to a new interactome). In this case, HNA could function as an inhibitor or an allosteric activator.

This mode of action is not amenable to Localis-REX.^45–47^ It would be applicable to study using photo-cross-linkers,^58^ for instance.

(iii) HNA is further metabolized

Another possibility is that HNA is further metabolized. Metabolism could create a new reactive species such as potentially an acylCoA or other activated acyl-containing molecule. These species show significantly different reactivity (ultimately labeling lysine, for instance) and likely locale of production than HNE. Thus, it is very likely that the signaling properties of HNA would be significantly different from those of HNE. We note that Cyps have also been shown to act on HNA, converting it to 9-OH-HNA. The role of this molecule is not clear to us.

Using current REX strategies,^37,45,59^ the difference between (i) and (iii) both of which involve covalent labeling of targets, would be difficult to differentiate. However, we note that REX strategies have always functioned on the premise that reactive electrophiles can interconvert, potentially between different reactive forms (a process we refer to as changing hands of information). Hence, all our Localis-REX studies have examined HNEylated proteins labeled minutes after the peak of HNE release. It has been our conjecture that, given that HNE labeling of thiols is overall slow and that metabolic processes also likely take some time, this allows us to focus on bona fide HNE sensors. As we have almost always been able to validate the labeling of our proteins by HNE, we believe that this is a fair assumption. Perhaps waiting longer, postphotouncaging or creating methods capable of releasing a lot more HNE than our current methods would also allow us to investigate these modes. In terms of the possibility of 9-OH-HNA formation being necessary for signaling, this postulate can be ruled out because we were able to show that terminally alkynylated HNA was able to signal appropriately.

(iv) HNA can affect lipid membrane biophysics or trafficking

As HNA is a lipophilic molecule, it could directly insert into membranes, changing their properties. There are many methods available to measure membrane biophysical properties in cells,^60–62^ although their applicability to *C elegans* is, as far as we are aware, yet to be disclosed. This could be studied in cell culture or in model systems as a first pass.

## Conclusion

Based on the above, we have outlined clear ways that apparently deactivated molecules, such as HNA, could nonetheless function to modulate flux through signaling pathways more efficiently than reactive counterparts, like HNE. We believe that all these mechanisms, perhaps in concert, will ultimately prove to be at work as more data are collected in this interesting area. Of course, we remain firm in our belief that HNE is a bona fide signaling molecule and that labeling of its protein targets is the main way HNE exerts its influence on cellular signaling. However, we remain convinced that some of HNE’s biological functions are a consequence of metabolic processes on HNE. These results clearly underscore the importance of performing better characterization of proposed HNE sensors, such as by defining phenotypes linked to on-target labeling, defining mutants that cannot be labeled by HNE, and deploying methods like T-REX to model HNEylation of specific target proteins.
